# Lifetime burden of disease due to incident tuberculosis: a global reappraisal including post-tuberculosis sequelae

**DOI:** 10.1016/S2214-109X(21)00367-3

**Published:** 2021-11-16

**Authors:** Nicolas A Menzies, Matthew Quaife, Brian W Allwood, Anthony L Byrne, Anna K Coussens, Anthony D Harries, Florian M Marx, Jamilah Meghji, Debora Pedrazzoli, Joshua A Salomon, Sedona Sweeney, Sanne C van Kampen, Robert S Wallis, Rein M G J Houben, Ted Cohen

**Affiliations:** a Department of Global Health and Population, Harvard T H Chan School of Public Health, Boston, MA, USA; b Center for Health Decision Science, Harvard TH Chan School of Public Health, Boston, MA, USA; c TB Modelling Group, TB Centre, London School of Hygiene & Tropical Medicine, London, UK; d Department of Infectious Disease Epidemiology, London School of Hygiene & Tropical Medicine, London, UK; e Faculty of Infectious and Tropical Diseases, London School of Hygiene & Tropical Medicine, London, UK; f Faculty of Public Health and Policy, London School of Hygiene & Tropical Medicine, London, UK; g Division of Pulmonology, Department of Medicine, Stellenbosch University, Stellenbosch, South Africa; h DSI-NRF South African Centre of Excellence in Epidemiological Modelling and Analysis (SACEMA), Stellenbosch University, Stellenbosch, South Africa; i Heart Lung Clinic, St Vincent's Hospital, Sydney, NSW, Australia; j Faculty of Medicine, St Vincent's Clinical School, University of New South Wales, Sydney, NSW, Australia; k Socios En Salud Sucursal Peru, Partners In Health, Lima, Peru; l Infectious Diseases and Immune Defence Division, The Walter & Eliza Hall Institute of Medical Research, Parkville, VIC, Australia; m Wellcome Centre for Infectious Diseases Research in Africa, Institute of Infectious Disease and Molecular Medicine, University of Cape Town, Observatory WC, South Africa; n Department of Medical Biology, University of Melbourne, Parkville, VIC, Australia; o International Union Against Tuberculosis and Lung Disease, Paris, France; p Desmond Tutu TB Centre, Department of Paediatrics and Child Health, Faculty of Medicine and Health Sciences, Stellenbosch University, Cape Town, South Africa; q Department of Clinical Sciences, Liverpool School of Tropical Medicine, Liverpool, UK; r Department of Medicine, Stanford University, Palo Alto CA, USA; s Department of Public Health and Primary Care, Leiden University Medical Center, Leiden, Netherlands; t The Aurum Institute, Parktown, Johannesburg, South Africa; u Department of Medicine, Vanderbilt University Medical Center, Nashville, TN, USA; v Department of Medicine, Case Western Reserve University, Cleveland, OH, USA; w Department of Medicine, Rutgers University, Newark, NJ, USA; x Department of Epidemiology of Microbial Diseases, Yale School of Public Health, New Haven, CT, USA

## Abstract

**Background:**

Many individuals who survive tuberculosis disease face ongoing disability and elevated mortality risks. However, the impact of post-tuberculosis sequelae is generally omitted from policy analyses and disease burden estimates. We therefore estimated the global burden of tuberculosis, inclusive of post-tuberculosis morbidity and mortality.

**Methods:**

We constructed a hypothetical cohort of individuals developing tuberculosis in 2019, including pulmonary and extrapulmonary disease. We simulated lifetime health outcomes for this cohort, stratified by country, age, sex, HIV status, and treatment status. We used disability-adjusted life-years (DALYs) to summarise fatal and non-fatal health losses attributable to tuberculosis, during the disease episode and afterwards. We estimated post-tuberculosis mortality and morbidity based on the decreased lung function caused by pulmonary tuberculosis disease.

**Findings:**

Globally, we estimated 122 (95% uncertainty interval [UI] 98–151) million DALYs due to incident tuberculosis disease in 2019, with 58 (38–83) million DALYs attributed to post-tuberculosis sequelae, representing 47% (95% UI 37–57) of the total burden estimate. The increase in burden from post-tuberculosis varied substantially across countries and regions, driven largely by differences in estimated case fatality for the disease episode. We estimated 12·1 DALYs (95% UI 10·0–14·9) per incident tuberculosis case, of which 6·3 DALYs (5·6–7·0) were from the disease episode and 5·8 DALYs (3·8–8·3) were from post-tuberculosis. Per-case post-tuberculosis burden estimates were greater for younger individuals, and in countries with high incidence rates. The burden of post-tuberculosis was spread over the remaining lifetime of tuberculosis survivors, with almost a third of total DALYs (28%, 95% UI 23–34) accruing 15 or more years after incident tuberculosis.

**Interpretation:**

Post-tuberculosis sequelae add substantially to the overall disease burden caused by tuberculosis. This hitherto unquantified burden has been omitted from most previous policy analyses. Future policy analyses and burden estimates should take better account of post-tuberculosis, to avoid the potential misallocation of funding, political attention, and research effort resulting from continued neglect of this issue.

**Funding:**

National Institutes of Health.

## Introduction

Tuberculosis was estimated to cause 1·4 million deaths in 2019.^1^ Individuals who develop tuberculosis experience a range of symptoms, including fever, wasting, and cough. Although successful treatment prevents death, many tuberculosis survivors experience ongoing health problems following the disease episode, and there is increasing evidence of long-term disability and elevated mortality risks in this population.^2^

The term post-tuberculosis describes the range of pathological conditions experienced by tuberculosis survivors. Pulmonary tuberculosis, the commonest disease presentation, causes progressive destruction of lung tissue, and this damage might not fully resolve after treatment.^3^, ^4^, ^5^ Although prompt treatment can minimise lung damage, many tuberculosis survivors experience residual lung pathology, with cross-sectional studies consistently demonstrating substantial pulmonary impairment—including chronic obstructive pulmonary disease (COPD), spirometric restriction, bronchiectasis, and pulmonary hypertension, as well as secondary non-tuberculosis lung infections—among tuberculosis survivors.^3^, ^4^, ^5^ These results are consistent with longitudinal evidence comparing lung function before and after the tuberculosis episode.^6^, ^7^ Individuals with chronic respiratory disease experience lower quality of life,^8^ higher health-care use,^9^ and reduced economic productivity compared with healthy individuals.^10^ Individuals with chronic respiratory disease additionally face higher all-cause mortality, even with only mild lung impairment.^2^, ^11^ In addition to pulmonary damage, extrapulmonary tuberculosis disease and drug toxicity can cause permanent damage to other organ systems, as well as social and psychological sequelae.^5^

**Research in context**

**Evidence before this study**

A growing number of cross-sectional and longitudinal studies have described chronic lung disease, long-term disability, and elevated mortality among individuals surviving tuberculosis disease. However, these post-tuberculosis sequelae are omitted from disease burden estimations and policy analyses that quantify the health losses caused by tuberculosis.

**Added value of this study**

We quantified the global burden of disease caused by incident tuberculosis in 2019, inclusive of post-tuberculosis morbidity and mortality over the lifetime of tuberculosis survivors. The results of this analysis demonstrate that post-tuberculosis burden adds considerably to the health losses caused by incident tuberculosis—globally, we estimated 122 (95% uncertainty interval 98–151) million disability-adjusted life-years (DALYs) due to tuberculosis occurring in 2019, with 58 (38–83) million DALYs attributed to post-tuberculosis sequelae, representing 47% (37–57) of the total burden estimate.

**Implications of all the available evidence**

When post-tuberculosis sequelae are considered, the disease burden caused by tuberculosis is substantially greater than conventional tuberculosis burden estimates. Accounting for post-tuberculosis in burden estimates and policy analyses will probably lead to revisions in the estimated impact of tuberculosis control efforts.

Historically, post-tuberculosis has not been a major focus of the tuberculosis control agenda. However, the health-care needs of tuberculosis survivors are drawing increasing attention,^5^ and recent modelling analyses have highlighted the large population of tuberculosis survivors, estimated at 155 million in 2020.^12^ The mortality and morbidity caused by post-tuberculosis have not traditionally been included in estimates of tuberculosis disease burden,^13^ nor policy analyses of tuberculosis control interventions.^14^ For burden estimates, with health losses quantified as disability-adjusted life-years (DALYs), health outcomes are attributed to specific diseases using the International Classification of Diseases (ICD) framework. Although ICD-10 includes a code for tuberculosis sequelae (B90.9), this code is rarely used. In practice, deaths among individuals with post-tuberculosis are commonly attributed to their proximal cause. For this reason, contemporary estimates of tuberculosis burden only include deaths and disability occurring during the tuberculosis disease episode.

Disease burden estimates shape the health research agenda and funding landscape, and they also determine how interventions are prioritised within disease control budgets. By excluding post-tuberculosis, current burden estimates might substantially underestimate the health losses caused by tuberculosis, which could lead to misallocation of funding, political attention, and research effort. Burden estimates for individual settings have provided initial evidence of the biases from omitting post-tuberculosis burden: an analysis estimating tuberculosis burden for India with and without post-tuberculosis found that total DALYs increased by 62% when post-tuberculosis was included.^15^ A study of tuberculosis burden in Tarrant County, TX, USA, found a similarly large fraction of total burden due to chronic tuberculosis sequelae.^16^ In this study, we estimated the total DALYs caused globally by incident tuberculosis disease in 2019, including attributable deaths and long-term disability among tuberculosis survivors in subsequent years. We report estimates for each of 186 countries reporting ten or more tuberculosis cases in 2019, to provide a more comprehensive description of the health losses caused by tuberculosis.

## Methods

### Study design

We constructed a hypothetical cohort of individuals developing tuberculosis in 2019, including pulmonary and extrapulmonary disease, who were stratified by factors related to the burden of tuberculosis or consequences after tuberculosis cure, and for whom sufficient data were available (country, age, sex, HIV status, and whether the individuals received tuberculosis treatment). We synthesised evidence describing quality of life and mortality among individuals with tuberculosis, during the disease episode and over their remaining life. Using this evidence, we simulated lifetime health outcomes for the cohort developing tuberculosis disease in 2019. We used DALYs to summarise the total health losses attributable to tuberculosis.

The WHO Global TB Programme reports annual estimates of the population developing tuberculosis disease, stratified by country, sex, and age group.^1^ For each country and sex, we interpolated these values to estimate incidence by single year of age in 2019. The fraction receiving treatment (by country, sex, and age) was based on reported case notifications^1^ divided by estimated incidence. For countries with missing notification data, we used WHO-estimated treatment coverage.^1^ We removed countries with insufficient data or with less than ten estimated tuberculosis cases for 2019. Applying these criteria, we retained 186 countries, representing 10·0 million tuberculosis cases, 99·99% of global incidence. To stratify cases by HIV status, we took estimates of age-specific HIV prevalence in the general population^17^ and inflated these by a common odds ratio (OR), to match the reported number of tuberculosis-HIV cases in each country.^1^ For countries for which this value was missing, we assumed tuberculosis-HIV prevalence was 0%. For each age and sex, the fraction receiving tuberculosis treatment was assumed to be independent of HIV status. We did not stratify the cohort by the presence or absence of multidrug-resistant tuberculosis (MDR-TB), extrapulmonary disease, or other factors that influence the severity of post-tuberculosis sequelae. The appendix (p 2) shows the distribution of the cohort across modelled strata.

### Mortality and disability weight estimates during and after tuberculosis

We adopted WHO estimates of tuberculosis case fatality in 2019.^1^ To apply these average mortality risks to individual strata, we specified ORs describing differences in survival by age (using age-specific data on tuberculosis cases and deaths in the USA^18^, ^19^), and by HIV and tuberculosis treatment status, based on mortality risks used in WHO tuberculosis estimations.^20^ We applied these ORs to each country and calibrated average mortality risks to reproduce WHO country-level case fatality estimates. The appendix (p 2) shows the fraction of the cohort estimated to survive the disease episode, by age.

Tuberculosis disability weights were based on current Global Burden of Disease Study (GBD) valuations^21^ (table 1). For HIV-uninfected individuals without tuberculosis we assumed no disability. For HIV-infected individuals without tuberculosis we averaged the disability weights for “HIV: symptomatic, pre-AIDS” and “HIV/AIDS: receiving antiretroviral treatment”, weighted by the global fraction of HIV-infected individuals receiving antiretroviral treatment in 2019 (67%).^17^ We calculated the incremental disability weight associated with tuberculosis disease as the difference between individuals with and without tuberculosis disease, stratified by HIV status. We assumed disability values applied for the duration of the disease episode, and adopted WHO disease duration estimates,^20^ stratified by treatment and HIV status (table 1).

**Table 1:** Values and sources for model parameters included in the uncertainty analysis

	Mean value (95% UI)*	**Prior distribution**	**Source**
Disability weight for tuberculosis	0·333 (0·224 to 0·454)	Beta	Global Burden of Disease Study (2019)^21^
Disability weight for tuberculosis and HIV	0·408 (0·274 to 0·549)	Beta	Global Burden of Disease Study (2019)^21^
Disability weight for HIV on ART†	0·078 (0·052 to 0·111)	Beta	Global Burden of Disease Study (2019)^21^
Disability weight for symptomatic HIV, no ART†	0·274 (0·184 to 0·377)	Beta	Global Burden of Disease Study (2019)^21^
Disability weight for mild COPD	0·019 (0·011 to 0·033)	Beta	Global Burden of Disease Study (2019)^21^
Disability weight for moderate COPD	0·225 (0·153 to 0·31)	Beta	Global Burden of Disease Study (2019)^21^
Disability weight for severe COPD	0·408 (0·273 to 0·556)	Beta	Global Burden of Disease Study (2019)^21^
Mortality rate ratio for post-tuberculosis individuals	2·91 (2·21 to 3·84)	Gamma	Romanowski et al (2019)^2^
Linear term for log-linear model for mortality by FEV_1_%	−2·783 (−3·908 to 1·662)	Normal	Duong et al (2019)^11^ (function fitted to study data)
Quadratic term for log-linear model for mortality by FEV_1_%	0·8947 (0·2759 to 1·522)	Normal	Duong et al (2019)^11^ (function fitted to study data)
Mortality risk ratio for post-tuberculosis (alternative specification)	1·78 (1·61 to 1·98)	Gamma	Lee-Rodriguez et al (2020)^22^
Intercept term for log-linear model for OR of chronic respiratory disease with post-tuberculosis	−1·494 (−3·223 to 0·321)	Normal	Byrne et al (2015)^23^ (function fitted to study data)
Slope term for log-linear model for OR of chronic respiratory disease in post-tuberculosis	0·45 (0·057 to 0·831)	Normal	Byrne et al (2015)^23^ (function fitted to study data)
Duration of treated tuberculosis	1·1 (0·2 to 2·0)	Gamma	WHO Global TB Database^1^
Duration of untreated tuberculosis	2·5 (1·0 to 4·0)	Gamma	WHO Global TB Database^1^
Duration of treated tuberculosis, with HIV	0·51 (0·01 to 1·0)	Gamma	WHO Global TB Database^1^
Duration of untreated tuberculosis, with HIV	0·11 (0·01 to 0·2)	Gamma	WHO Global TB Database^1^
Total global tuberculosis cases in 2019	9·96 (8·94 to 11·00)	Gamma	WHO Global TB Database^1^
Global average tuberculosis case fatality rate in 2019	0·14 (0·13 to 0·16)	Beta	WHO Global TB Database^1^
Fraction of tuberculosis cases untreated	0·29 (0·20 to 0·35)	Beta	WHO Global TB Database^1^

ART=antiretroviral therapy. COPD=chronic obstructive pulmonary disease. FEV_1_%=forced expiratory volume in 1 s. OR=odds ratio. UI=uncertainty interval.

* Lower and upper bounds represent the 2·5th and 97·5th percentiles of the parameter distribution. Together these represent an equal-tailed 95% UI for the parameter.

† Disability weights for HIV without tuberculosis are applied to HIV-infected tuberculosis survivors.

Individuals surviving tuberculosis disease face elevated mortality risks. A recent meta-analysis reported a standardised mortality ratio of 2·91 (95% CI 2·21–3·84) for tuberculosis survivors, compared with individuals without previous tuberculosis.^2^ These elevated risks reflect a combination of the causal impact of tuberculosis on future mortality risks and the effect of individual characteristics that are correlated with both mortality rates and tuberculosis disease, but not caused by tuberculosis. To decompose these two effects, we assumed that the causal effect of tuberculosis on future mortality can be estimated from the effects of tuberculosis on lung function, and the resulting effects of impaired lung function on mortality rates.

Two systematic reviews have assessed the elevated rates of chronic respiratory disease among tuberculosis survivors.^23^, ^24^ The more recent of these reviews described an association between the odds of COPD among individuals with post-tuberculosis and country-level tuberculosis incidence. In this context, incidence is probably a proxy for delayed case detection, recurrent disease episodes, and other risk factors for severe disease.^5^ We estimated a meta-regression model summarising this association (appendix p 3) and used these results to estimate an OR of chronic respiratory disease among individuals with post-tuberculosis for each country, based on their 2019 tuberculosis incidence (appendix p 4). We used the results of a multisite observational study^11^ to describe the population distribution of lung function, quantified as country-standardised forced expiratory volume in 1 s (FEV_1_%), and assumed that tuberculosis induced a downward shift of this distribution. For each country, the reduction in FEV_1_% among tuberculosis survivors was estimated to match the ORs for chronic respiratory disease in tuberculosis survivors, which was defined as an FEV_1_% lower than 80%. To model the association between FEV_1_% and mortality we fitted a quadratic function to mortality rate ratios (RRs) reported for different FEV_1_% impairment levels,^11^ and estimated the mortality risk ratio for post-tuberculosis as the average mortality rate for the post-tuberculosis FEV_1_% distribution compared with the distribution without post-tuberculosis. Country-level mortality RRs estimated for post-tuberculosis varied between 1·02 and 1·34, with a median of 1·14 (appendix p 4), and they were applied to all modelled strata.

We estimated all-cause mortality rates for each country, sex, age, and calendar year, by interpolating UN Population Division abridged life tables.^25^ To account for excess mortality among HIV-positive individuals we assumed a mortality RR producing an 8-year shorter life expectancy for HIV-positive individuals than for HIV-negative individuals.^26^ To calculate future mortality rates for tuberculosis survivors we multiplied all-cause general population mortality rates for each stratum by the standardised mortality ratio reported for tuberculosis survivors.^2^ To calculate future mortality rates for the cohort under a counterfactual where they had not developed tuberculosis, we divided the mortality rates estimated for the tuberculosis scenario by the causal mortality RR estimated for each country.

We adapted COPD disability weights to account for the reduced quality of life of tuberculosis survivors.^21^ We adopted a mapping between COPD severity levels and FEV1% impairment level from an earlier study of COPD burden,^8^ and applied these disability weights to the distribution of tuberculosis survivors across FEV_1_% impairment levels to calculate a post-tuberculosis disability weight for each country. Country-level estimates of the incremental disability caused by post-tuberculosis varied between 0·006 and 0·088, with a median of 0·036 (appendix p 4). These were applied to all model strata over the remaining life expectancy.

### Statistical analysis

The primary outcome was DALYs attributable to tuberculosis, stratified by tuberculosis disease and post-tuberculosis periods, using a lifetime horizon. DALYs are calculated as the sum of years of life lost (YLLs) and years lived with disability (YLDs).^27^ We computed YLLs by multiplying deaths at each age by age-specific life expectancy, based on the GBD reference life table.^28^ We computed YLDs by multiplying total years lived in each health state by the disability weight for that state. As DALYs arising during the tuberculosis disease episode are also reported by the GBD study,^29^ we validated our results for this outcome against these existing estimates. As a secondary outcome, we calculated the reductions in life expectancy due to tuberculosis disease and post-tuberculosis compared with a no-tuberculosis counterfactual.

We specified probability distributions to represent uncertainty in model parameters (table 1), and we used second-order Monte Carlo simulation to generate 95% uncertainty intervals (UIs) for study outcomes.^30^ To do so, we re-estimated results for 1000 Latin hypercube samples from the parameter probability distributions, and calculated intervals as the 2·5th and 97·5th percentiles of the resulting distributions of each outcome. Analyses were conducted in R (v4.0.2).^31^

The mortality RR for post-tuberculosis used in the main analysis is conservative, as it only captures one of several mechanisms through which tuberculosis affects future mortality risks. As a sensitivity analysis, we recalculated results using a mortality risk ratio for post-tuberculosis of 1·78 (95% CI 1·61–1·98), based on a retrospective cohort study of individuals with and without post-tuberculosis, controlling for multiple demographic and clinical risk factors for mortality.^22^ We also calculated partial rank correlation coefficients (PRCCs) to report the relative influence of individual parameters on study outcomes.^32^

### Role of the funding source

The funder of the study had no role in study design, data collection, data analysis, data interpretation, or writing of the report.

## Results

Table 2 shows estimates of the total DALYs attributable to incident tuberculosis cases from 2019. Globally, we estimated 122 (95% UI 98–151) million DALYs, with 64 (54–75) million attributed to the tuberculosis disease episode, and 58 (38–83) million attributed to post-tuberculosis, representing 47% (37–57) of the total burden estimate. Globally, DALY estimates for the tuberculosis disease episode were 8·3% higher than GBD estimates (appendix p 5), largely due to greater tuberculosis mortality estimated by WHO^1^ than that estimated in the GBD study.^29^ Results for individual countries were highly consistent (rank correlation 0·97, appendix p 5). Mortality (reflected in the YLL results) represented the large majority (93% [88–96]) of DALYs from the tuberculosis disease episode.

**Table 2:** Burden of disease caused by incident tuberculosis in 2019, globally and by WHO region

	**Outcomes by disease stage (millions)**			Percent increase with post-tuberculosis (%)*
	Tuberculosis disease	Post-tuberculosis	Total	
**Years of life lost**				
Eastern Mediterranean	2·52 (2·14–2·99)	3·55 (2·38–5·01)	6·07 (4·78–7·68)	141% (95–206)
Europe	0·88 (0·75–1·02)	0·64 (0·41–0·93)	1·51 (1·23–1·83)	73% (48–109)
Africa	25·35 (21·51–30·03)	12·46 (8·09–18·11)	37·81 (31·49–45·26)	49% (32–73)
Western Pacific	2·87 (2·45–3·36)	6·91 (4·70–9·75)	9·78 (7·49–12·68)	242% (164–351)
Americas	0·75 (0·65–0·88)	0·75 (0·50–1·08)	1·51 (1·21–1·86)	100% (66–148)
Southeast Asia	26·90 (23·06–31·50)	17·81 (11·92–25·38)	44·72 (37·02–53·84)	66% (44–97)
WHO high-burden	50·96 (43·47–59·89)	37·52 (24·91–53·46)	88·48 (72·75–107·52)	74% (49–109)
Global	59·27 (50·56–69·63)	42·12 (28·32–59·77)	101·39 (83·64–122·78)	71% (47–104)
**Years lived with disability**				
Eastern Mediterranean	0·44 (0·22–0·77)	1·36 (0·75–2·13)	1·80 (1·13–2·63)	340% (142–686)
Europe	0·10 (0·04–0·19)	0·21 (0·12–0·35)	0·31 (0·19–0·49)	262% (96–596)
Africa	1·13 (0·57–1·94)	3·43 (1·80–5·44)	4·56 (2·83–6·65)	331% (136–659)
Western Pacific	0·78 (0·36–1·44)	2·75 (1·54–4·28)	3·53 (2·20–5·18)	394% (153–809)
Americas	0·11 (0·05–0·21)	0·33 (0·19–0·52)	0·44 (0·28–0·67)	327% (126–691)
Southeast Asia	1·96 (0·92–3·52)	7·57 (4·13–11·99)	9·53 (5·73–14·04)	432% (167–886)
WHO high-burden	3·91 (1·90–6·93)	14·02 (7·65–22·14)	17·94 (10·90–26·25)	397% (156–789)
Global	4·53 (2·21–8·00)	15·64 (8·61–24·43)	20·17 (12·48–29·52)	383% (154–755)
**Disability-adjusted life-years**				
Eastern Mediterranean	2·96 (2·47–3·54)	4·90 (3·27–7·02)	7·87 (6·05–10·21)	167% (106–244)
Europe	0·97 (0·82–1·14)	0·85 (0·55–1·26)	1·82 (1·45–2·27)	88% (56–134)
Africa	26·48 (22·46–31·29)	15·89 (10·19–23·31)	42·37 (35·01–51·52)	60% (38–90)
Western Pacific	3·65 (2·98–4·50)	9·66 (6·50–13·84)	13·31 (9·89–17·63)	267% (169–388)
Americas	0·87 (0·73–1·03)	1·08 (0·71–1·58)	1·94 (1·51–2·49)	125% (82–186)
Southeast Asia	28·87 (24·47–33·66)	25·38 (16·67–36·84)	54·25 (43·87–67·66)	88% (56–130)
WHO high-burden	54·88 (46·34–64·69)	51·54 (33·78–74·85)	106·42 (85·74–132·97)	94% (61–140)
Global	63·79 (53·87–75·16)	57·76 (38·37–82·97)	1121·55 (98·29–151·19)	91% (59–133)

Values in parentheses represent 95% uncertainty intervals.

* Compares outcomes with *vs* without the inclusion of post-tuberculosis.

DALYs attributable to post-tuberculosis have not been included in past tuberculosis burden estimates, and their inclusion substantially increases burden estimates, by 91% (95% UI 59–132) globally. The increase in total DALYs produced by considering post-tuberculosis varied substantially by region, from 60% in Africa to 267% in the Western Pacific. The proportional increase in YLDs (reflecting the burden of non-fatal morbidity) was 383% (154–755), reflecting the extended duration of post-tuberculosis relative to the tuberculosis disease episode. The proportional increase in YLLs was 71% (47–104). Reductions in survival represent the majority (73% [68–79]) of DALYs from the post-tuberculosis period. We also estimated the DALYs from tuberculosis disease and post-tuberculosis for the 30 high-tuberculosis-burden countries identified by WHO (appendix p 8). In these results, the percent increase in DALYs resulting from the inclusion of post-tuberculosis varied widely between countries, driven largely by differences in WHO-estimated tuberculosis case fatality (appendix p 6), rank correlation= –0·74). When we looked at the global distribution of post-tuberculosis DALYs (appendix p 7), there were 7·5 (5·0–10·8) post-tuberculosis DALYs per 1000 individuals globally, and 21 countries with more than 20 post-tuberculosis DALYs per 1000 individuals.

**Figure 1:**
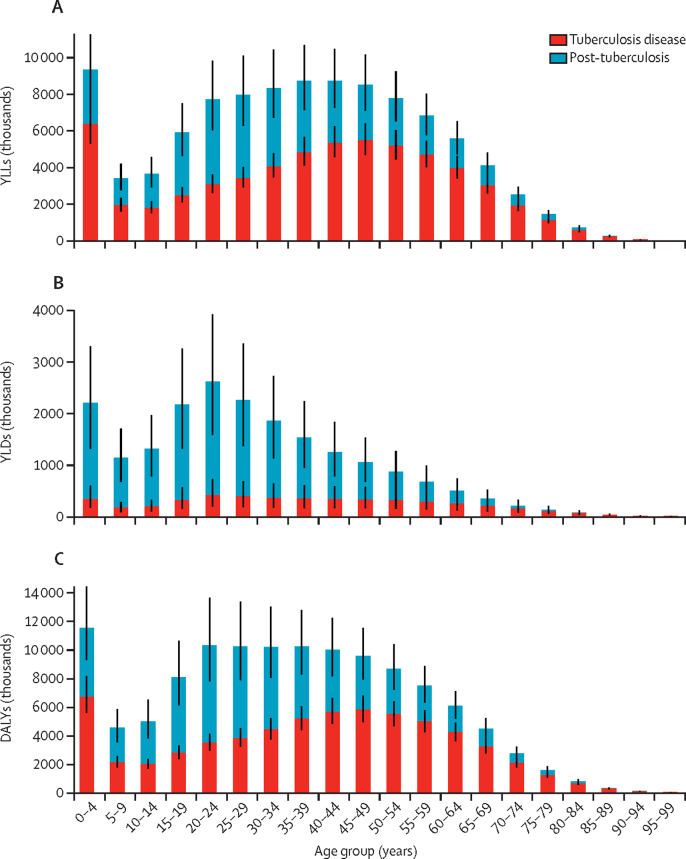
Estimates of YLLs (A), YLDs (B), and DALYs (C) attributable to tuberculosis disease in 2019, stratified by age group of disease incidence, and disease period* YLL=years of life lost. YLD=years lived with disability. DALYs=disability-adjusted life-years. *Black bars represent 95% uncertainty intervals.

Figure 1 shows global estimates for YLLs, YLDs, and DALYs stratified by age, showing the increase in burden associated with post-tuberculosis in each age group. The proportional increase in DALYs from post-tuberculosis was smaller in older age groups, reflecting higher fatality during tuberculosis disease among older individuals.

**Figure 2:**
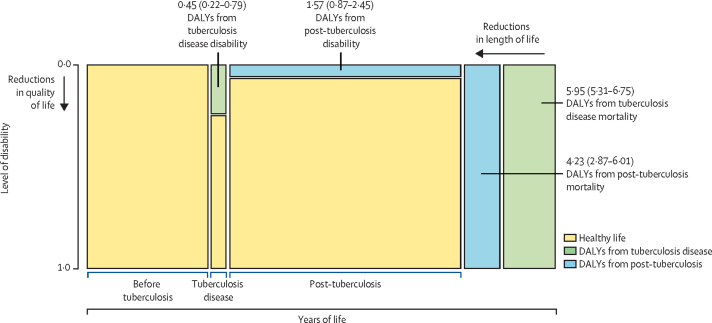
Average DALYs per incident tuberculosis case from increased disability and mortality rates attributable to tuberculosis, stratified by tuberculosis disease and post-tuberculosis period* Area of each green and blue rectangle is proportional to the number of DALYs indicated, other dimensions not to scale. Values in parentheses represent 95% uncertainty intervals. DALYs=disability-adjusted life-years. *Total DALYs per incident tuberculosis case are equal to the sum of these values.

Figure 2 shows a schematic of the contribution of mortality and disability to the average global DALYs per individual developing tuberculosis in 2019, from both the tuberculosis disease episode and post-tuberculosis period. Globally, we estimated 12·1 DALYs (95% UI 10·0–14·9) per incident tuberculosis case, of which 6·3 DALYS (5·6–7·0) were attributable to the disease episode and 5·8 DALYS (3·8–8·3) attributable to post-tuberculosis.

Total and per-person DALY estimates varied by individual-level and country-level factors (table 3). The average DALYs per incident case were estimated to be higher for younger individuals, HIV-positive individuals, individuals who do not receive tuberculosis treatment, and individuals living in high-incidence countries.

**Table 3:** Total and per-person estimates of the burden of disease caused by incident tuberculosis in 2019

		**Total tuberculosis cases (thousands)**	**Absolute DALYs (millions)**			**DALYs per incident tuberculosis case**		
			Tuberculosis disease	Post-tuberculosis	Total	Tuberculosis disease	Post-tuberculosis	Total
All		9954 (8952–11 003)	63·79 (53·87–75·16)	57·76 (38·37–82·97)	121·55 (98·29–151·19)	6·41 (5·69–7·20)	5·80 (3·83–8·34)	12·21 (10·02–14·85)
Age group								
	1–4 years	1185 (1065–1309)	10·75 (8·97–13·02)	10·26 (6·51–15·03)	21·00 (16·61–26·56)	9·07 (7·92–10·80)	8·66 (5·52–12·92)	17·73 (14·16–21·99)
	15–34 years	3307 (2974–3655)	14·43 (12·16–17·06)	24·32 (16·14–35·07)	38·75 (29·76–50·70)	4·36 (3·85–4·97)	7·36 (4·82–10·71)	11·72 (9·12–15·03)
	35–54 years	3039 (2733–3359)	22·06 (18·72–25·77)	16·32 (10·85–23·19)	38·37 (31·55–46·87)	7·26 (6·47–8·18)	5·37 (3·59–7·65)	12·63 (10·61–15·02)
	55–74 years	1998 (1797–2208)	14·41 (12·26–16·76)	6·29 (4·27–9·00)	20·69 (17·61–24·29)	7·21 (6·41–8·01)	3·15 (2·17–4·45)	10·36 (9·09–11·79)
	≥75 years	426 (383–471)	2·15 (1·83–2·51)	0·58 (0·40–0·83)	2·73 (2·33–3·19)	5·05 (4·47–5·65)	1·36 (0·95–1·92)	6·41 (5·77–7·17)
Sex								
	Male	6158 (5538–6807)	39·75 (33·58–46·81)	35·61 (23·69–51·05)	75·36 (61·12–93·50)	6·45 (5·73–7·24)	5·78 (3·85–8·26)	12·24 (10·10–14·82)
	Female	3796 (3414–4196)	24·04 (20·29–28·35)	22·15 (14·63–32·05)	46·19 (37·13–57·77)	6·33 (5·62–7·13)	5·84 (3·81–8·50)	12·17 (9·93–14·84)
Tuberculosis incidence level (cases per 100 000 population)								
	<10	51 (46–56)	0·13 (0·11–0·15)	0·08 (0·03–0·18)	0·21 (0·15–0·31)	2·48 (2·15–2·87)	1·60 (0·50–3·68)	4·08 (2·93–6·30)
	10–49	331 (298–366)	1·05 (0·89–1·25)	1·02 (0·58–1·58)	2·07 (1·58–2·69)	3·19 (2·78–3·62)	3·08 (1·82–4·84)	6·27 (4·92–8·02)
	50–199	4726 (4250–5224)	29·43 (24·90–34·33)	23·70 (16·26–34·37)	53·13 (43·57–64·51)	6·23 (5·53–6·95)	5·02 (3·47–7·10)	11·24 (9·53–13·53)
	≥200	4846 (4359–5357)	33·19 (28·02–39·07)	32·96 (20·25–49·02)	66·14 (52·32–83·97)	6·85 (6·05–7·81)	6·80 (4·09–10·14)	13·65 (10·79–17·05)
HIV status								
	HIV positive	999 (899–1104)	11·84 (10·12–13·84)	5·81 (3·78–8·44)	17·65 (14·84–21·04)	11·85 (10·59–13·39)	5·82 (3·79–8·51)	17·67 (15·27–20·45)
	HIV negative	8955 (8053–9898)	51·95 (43·86–61·16)	51·95 (34·55–74·36)	103·90 (83·26–130·27)	5·80 (5·13–6·54)	5·80 (3·83–8·36)	11·60 (9·44–14·24)
Tuberculosis treatment								
	Treated	7224 (6230–8270)	17·43 (9·75–28·92)	43·84 (28·17–64·45)	61·27 (40·87–86·32)	2·39 (1·48–3·59)	6·07 (4·09–8·76)	8·46 (6·10–11·35)
	Untreated	2730 (1999–3530)	46·36 (33·70–60·53)	13·92 (8·04–22·31)	60·28 (43·59–79·15)	16·99 (15·24–18·81)	5·10 (3·27–7·55)	22·09 (19·51–24·84)

Values in parentheses represent 95% uncertainty intervals. DALYs=disability-adjusted life-years.

**Figure 3:**
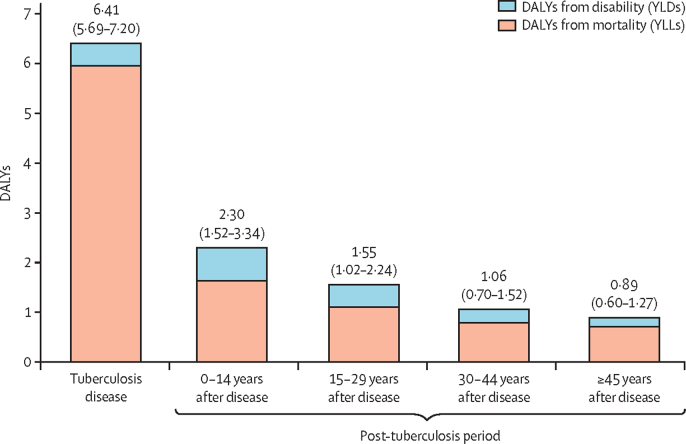
DALYs per incident tuberculosis case, stratified by tuberculosis disease and post-tuberculosis period* YLL=years of life lost. YLD=years lived with disability. DALYs=disability-adjusted life-years. *Total DALYs per incident tuberculosis case equal to the sum of these values. Values in parentheses represent 95% uncertainty intervals.

Figure 3 shows the average DALYs per case according to when the burden (ie, the tuberculosis-attributable death, or time spent with disability) occurs. The tuberculosis disease episode represented a short period of high disability and mortality. By contrast, the DALYs from post-tuberculosis were spread over the remaining lifespan, with almost a third (28% [23–34]) of total DALYs accruing 15 or more years after an individual first developed tuberculosis.

The appendix (p 9) shows reductions in life-years lived due to tuberculosis disease and post-tuberculosis, compared with a counterfactual in which individuals do not develop tuberculosis. Per incident tuberculosis case, we estimated an average reduction in life expectancy from mortality during the tuberculosis disease episode of 2·92 years (2·60–3·35) and an additional reduction in life expectancy from post-tuberculosis mortality of 1·87 years (1·21–2·80). The average reduction in life expectancy attributed to incident tuberculosis in 2019 (summing the effects of excess mortality over the life course) was 4·79 years (3·94–5·86).

The appendix (p 10) shows results for the alternative model specification with a higher mortality risk ratio for post-tuberculosis. In these results, total YLLs from post-tuberculosis increased from 42·1 (28·3–59·8) million to 94·1 (78·7–111·6) million. The appendix (p 11) shows PRCCs for each model parameter, for several outcomes. For DALYs due to the tuberculosis disease episode, the most influential parameters were the inputs for total global incidence in 2019 (PRCC=0·89), and case fatality during the disease episode (PRCC=0·89). For DALYs due to post-tuberculosis, the most influential parameters were the OR of respiratory disease with post-tuberculosis (PRCC=0·86), and total global incidence in 2019 (PRCC=0·46). For both total DALYs and the percentage increase in DALYs from post-tuberculosis, the most influential parameter was the OR of respiratory disease with post-tuberculosis (PRCC=0·83 and 0·85, respectively).

## Discussion

To our knowledge, this study is the first to estimate the global burden of tuberculosis inclusive of post-tuberculosis morbidity and mortality. Even with conservative assumptions, the inclusion of post-tuberculosis substantially increased the total burden estimate. For the 8·5 million individuals surviving incident tuberculosis in 2019, we estimated that 58 million post-tuberculosis DALYs will accrue over the remaining lifetime, representing almost half of the 122 million DALYs estimated for tuberculosis.

Our burden estimate was approximately twice the GBD estimate for tuberculosis DALYs in the same year.^29^ Our results do not represent a proposed correction of the GBD estimates, as the two approaches answer different questions. First, we adopted an incidence approach, quantifying DALYs that would accrue over the lifetime due to disease cases arising in a single year. By contrast, GBD uses a prevalence approach to estimate health losses from disability, summing the DALYs realised in a given year irrespective of when the causative disease case occurred. Second, and more fundamentally, the GBD estimates follow ICD disease classification conventions, with the consequence that incremental deaths due to post-tuberculosis are attributed to causes such as respiratory or cardiovascular disease, rather than tuberculosis. Within the GBD framework, the role of post-tuberculosis is more similar to a risk factor, as it increases the risks of other diseases over the lifetime.

The primary disease burden tabulations produced by GBD are calculated to be categorical, such that the sum of DALYs for each individual disease equals the total DALYs from all diseases. However, users of disease burden estimates commonly interpret them to be the burden caused by a given disease, in the sense that they summarise the death and disability avertable through global elimination of that disease. If our results are correct, the causal interpretation of current GBD estimates for tuberculosis disease burden substantially undervalues the potential impact of tuberculosis control efforts. Appropriate valuation of post-tuberculosis disease burden might also lead to reprioritisation within the tuberculosis control portfolio. This could include greater attention to programmatic activities that can prevent tuberculosis from occurring in the first place, to active case-finding that may accelerate diagnosis, and to interventions that can prevent, mitigate, or repair lung damage from tuberculosis. These changes are consistent with a broader definition of treatment success, beyond simply sterilising the causative pathogen. This could include a greater role for end-of-treatment assessments of pathological damage and functional deficits in order to implement interventions that meet the health-care needs of tuberculosis survivors, who in any given year represent a much larger group than the individuals newly developing tuberculosis.^12^ While initial assessment can be done by tuberculosis programmes, ongoing care will probably require integrated, multi-specialty services.^33^

The magnitude of post-tuberculosis disease burden also has implications for new research. In particular, further research is needed on the mechanisms of post-tuberculosis impairment, the range of conditions experienced by tuberculosis survivors, and how the extent of impairment changes over time.^34^ Research is also needed to describe management strategies for post-tuberculosis symptoms and disability, and their effectiveness at improving long-term outcomes for tuberculosis survivors. Policy analyses used to prioritise tuberculosis interventions should also take greater account of post-tuberculosis burden. This new approach may lead to a reweighting of current intervention priorities, and to the identification of a cost-effective portfolio of services for post-tuberculosis individuals. Finally, research is needed to better understand the social and economic implications of post-tuberculosis, including health-care use, social exclusion, and household economic outcomes. This is particularly important given the global target of achieving zero households experiencing catastrophic costs from tuberculosis, described in the End TB Strategy.^35^

Our analysis has several limitations. First, our results might represent an underestimate of post-tuberculosis burden, as we only considered one pathway through which tuberculosis affects future health outcomes. Tuberculosis disease has deleterious effects on multiple organ systems, and some tuberculosis survivors experience respiratory symptoms even in the absence of clinically impaired lung function.^36^ Chronic post-tuberculosis disability can also lead to stigma, social exclusion, and poverty, which themselves predispose to additional health risks. A more comprehensive assessment of these effects could produce a larger estimate of post-tuberculosis DALYs. As an indicator of what a more comprehensive assessment might imply, the alternative specification using a higher mortality risk ratio for post-tuberculosis increased the total tuberculosis burden estimate by a further 40%. Second, there is substantial uncertainty associated with our estimates, including parameter uncertainty (quantified as UIs around major outcomes), and structural uncertainty, which represents uncertainty about the functional form of the analytic model, not captured in the reported intervals. A major source of uncertainty is the weakness around the causal attribution of post-tuberculosis health outcomes to tuberculosis (as compared with existing risk factors among individuals developing tuberculosis). These causal assumptions affect the mortality risk ratios and disability weights for tuberculosis survivors. In our analyses, we estimated that the contribution of post-tuberculosis to elevated mortality among tuberculosis survivors^2^ varied between 2% and 27% across countries (median 12%), yet there is substantial uncertainty around the average value as well as intercountry differences. Age-specific ORs for tuberculosis mortality represent another source of uncertainty. Finally, our analysis did not distinguish conditions for which post-tuberculosis outcomes are likely to be different, including multidrug-resistant and extensively drug-resistant tuberculosis, recurrent tuberculosis, extrapulmonary tuberculosis, or extensive disease at initial presentation. For most of these conditions the burden of post-tuberculosis could be greater than estimated in this analysis.^34^ Similarly, outcomes might be different for children surviving tuberculosis, and better evidence on paediatric post-tuberculosis is needed.

Many individuals who survive tuberculosis disease face ongoing disability, despite achieving microbiological cure. For these tuberculosis survivors, post-tuberculosis sequelae limit opportunities and enjoyment of life, and increase the risk of early death. Our analysis has quantified these health losses, demonstrating that post-tuberculosis adds substantially to the global disease burden caused by tuberculosis. Future analyses should take better account of post-tuberculosis, to avoid the potential misallocation of funding, political attention, and research effort that would be produced by continued neglect of this issue.

## Data sharing

Analytic code and data inputs are available upon request to the corresponding author (nmenzies@hsph.harvard.edu).

## Declaration of interests

We declare no competing interests.
