# Correction to *Lancet Glob Health* 2021; 9: e1679–87

**DOI:** 10.1016/S2214-109X(22)00037-7

**Published:** 2022-02-15

*Menzies NA, Quaife M, Allwood BW, et al. Lifetime burden of disease due to incident tuberculosis: a global reappraisal including post-tuberculosis sequelae.* Lancet Glob Health *2021; **9:** e1679–87*—In this Article, the grant numbers for the funding from Horizon 2020 and EDCTP have been added to the acknowledgments section. This correction has been made as of Feb 15, 2022.

