# Supplementary material for: Lifetime burden of disease due to incident tuberculosis: a global reappraisal including post-tuberculosis sequelae
Source: Lancet Glob Health. 2021 Nov 16;9(12):e1679–87. doi: 10.1016/S2214-109X(21)00367-3 (PMC8609280; doi:10.1016/S2214-109X(21)00367-3)
Supplement: Supplementary appendix [file mmc1.pdf]

THE LANCET
Global Health

Supplementary appendix

This appendix formed part of the original submission and has been peer reviewed. We post it as supplied by the authors.

Supplement to: Menzies NA, Quaife M, Allwood BW, et al. Lifetime burden of disease due to incident tuberculosis: a global reappraisal including post-tuberculosis sequelae. *Lancet Glob Health* 2021; **9:** e1679–87.

Supplement: "The lifetime burden of disease due to incident tuberculosis: a global re-appraisal including post-TB sequelae."

Contents

Figure S1: Total individuals developing TB disease in 2019, stratified by age, sex, HIV,TB treatment status, and survival of the TB disease episode 2

Figure S2: Published estimates for the adjusted odds ratio of COPD among individuals surviving TB, compared to a matched control group, with results from a meta-regression fit to these study estimates ... 3

Figure S3: Distribution of country-level values estimated for the odds ratio of chronic respiratory disease (Panel A), the causal mortality risk-ratio (Panel B), and the disability weights (Panel C) estimated for post-TB individuals 4

Figure S4: Comparisons between burden of TB estimates at global level (Panel A) and country level (Panel B), from this analysis compared to results published by the Global Burden of Disease study for 2019 5

Figure S5: Country-level values for the case fatality of the TB disease episode plotted against the increase in total DALYs due to the inclusion of post-TB, log-log projection 6

Figure S6: Global distribution of post-TB DALYs per 1000, attributable to incident TB disease in 2019..... 7

Table S1: Estimated DALYs for TB disease and post-TB (millions), for the thirty high-TB burden countries, 2019 8

Table S2: Total reductions in life-years lived as a result of incident TB in 2019, globally and by world region 9

Table S3: Global burden of disease outcomes for TB disease and post-TB due to incident TB in 2019, comparing results from the main analysis an alternative specification with a higher mortality risk ratio for post-TB 10

Table S4: Partial rank correlation coefficients (PRCCs) for four major outcomes (global DALYs for TB disease, post-TB, and both combined, and the percentage increase in total DALYs with the inclusion of post-TB) 11

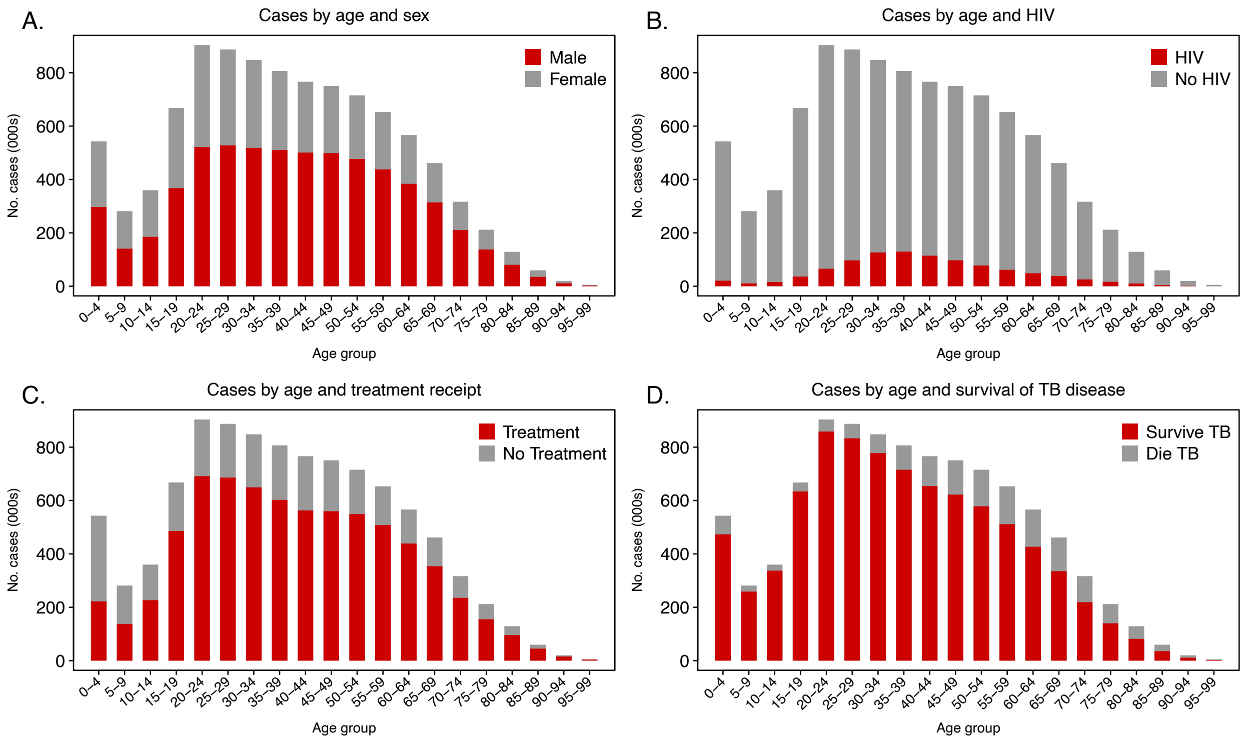

Figure S1: Total individuals developing TB disease in 2019, stratified by age, sex, HIV,TB treatment status, and survival of the TB disease episode*.

* Panel A: total TB cases in 2019 stratified by age and sex. Panel B: total TB cases in 2019 stratified by age and HIV status. Panel A: total TB cases in 2019 stratified by age and TB treatment status. Panel A: total TB cases in 2019 stratified by age and survival of the TB disease episode.

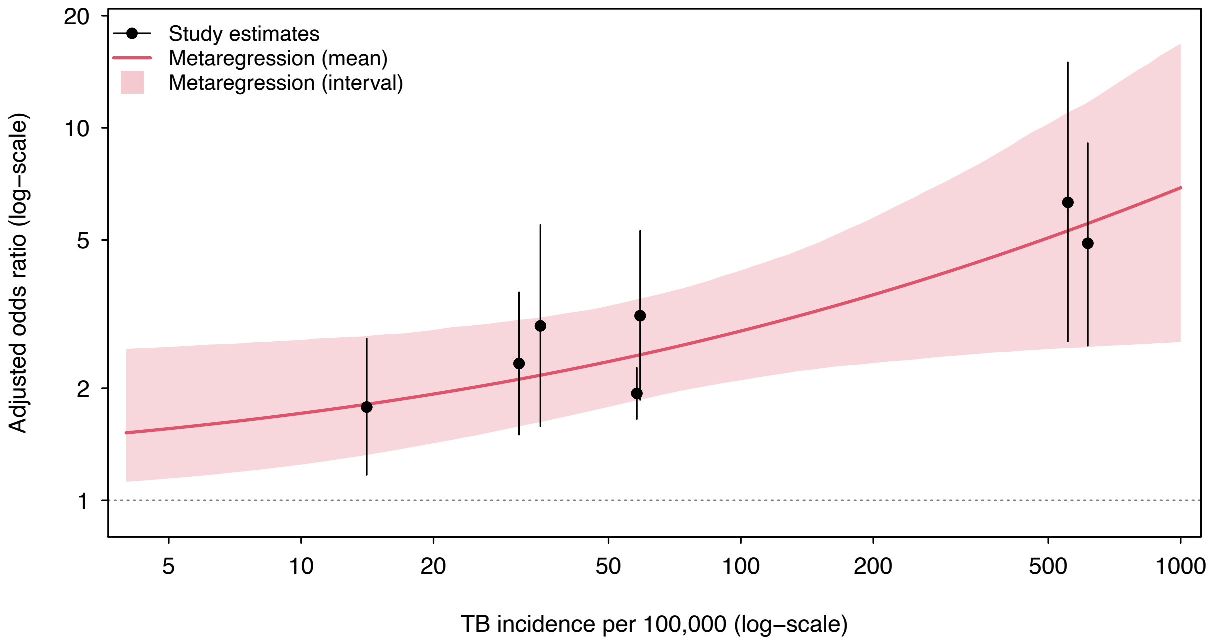

Figure S2: Published estimates for the adjusted odds ratio of COPD among individuals surviving TB, compared to a matched control group, with results from a meta-regression fit to these study estimates*.

* Based on results reported in Byrne et al, *Int J Infect Dis* 2015.

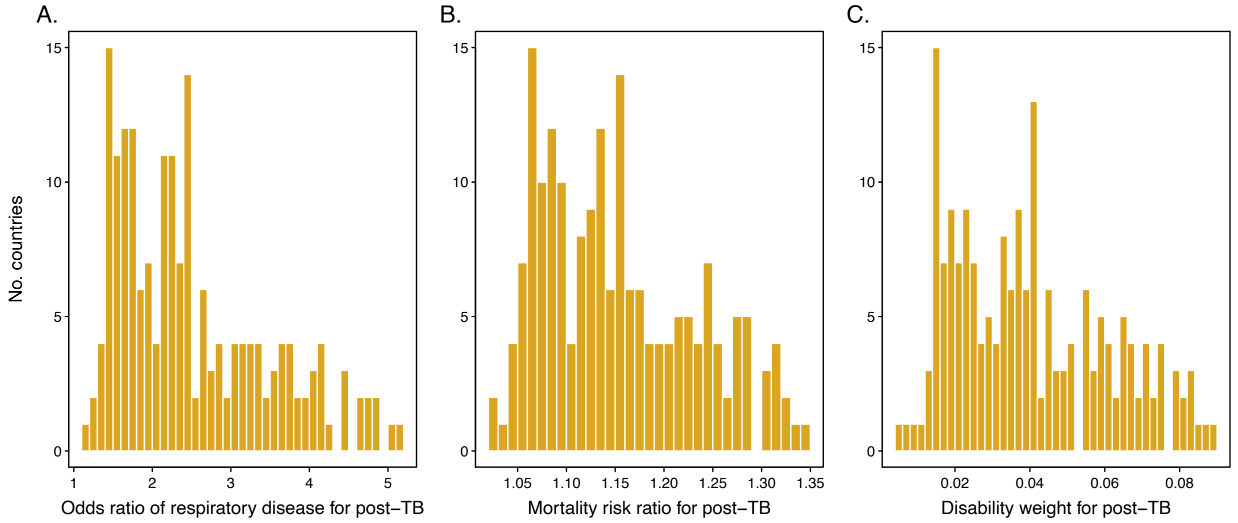

Figure S3: Distribution of country-level values estimated for the odds ratio of chronic respiratory disease (Panel A), the causal mortality risk-ratio (Panel B), and the disability weights (Panel C) estimated for post-TB individuals.

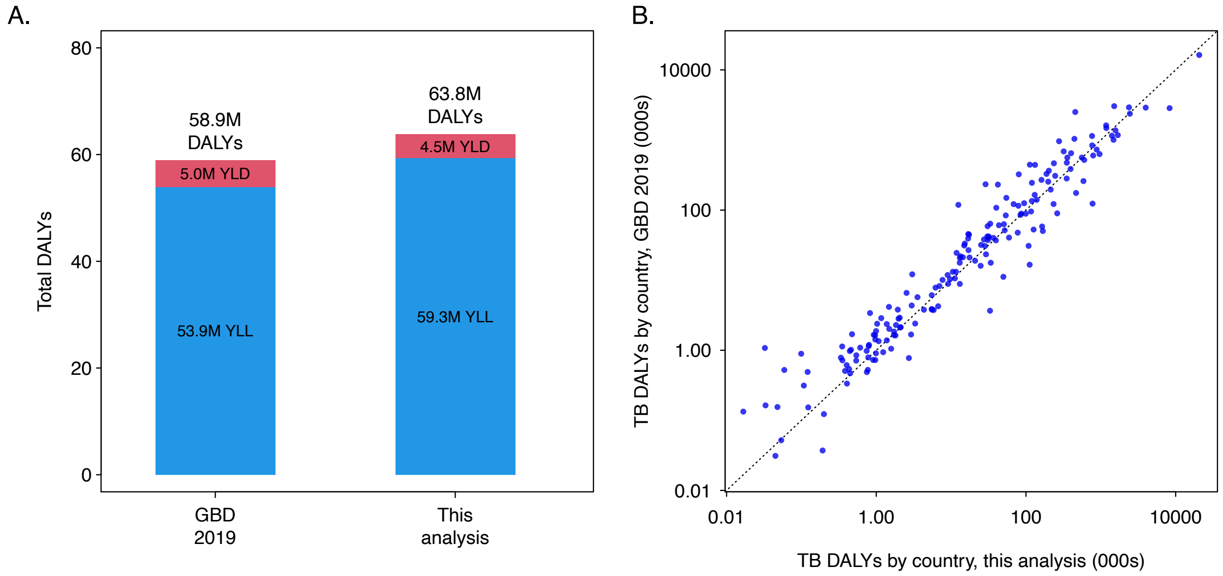

Figure S4: Comparisons between burden of TB estimates at global level (Panel A) and country level (Panel B), from this analysis compared to results published by the Global Burden of Disease study for 2019.

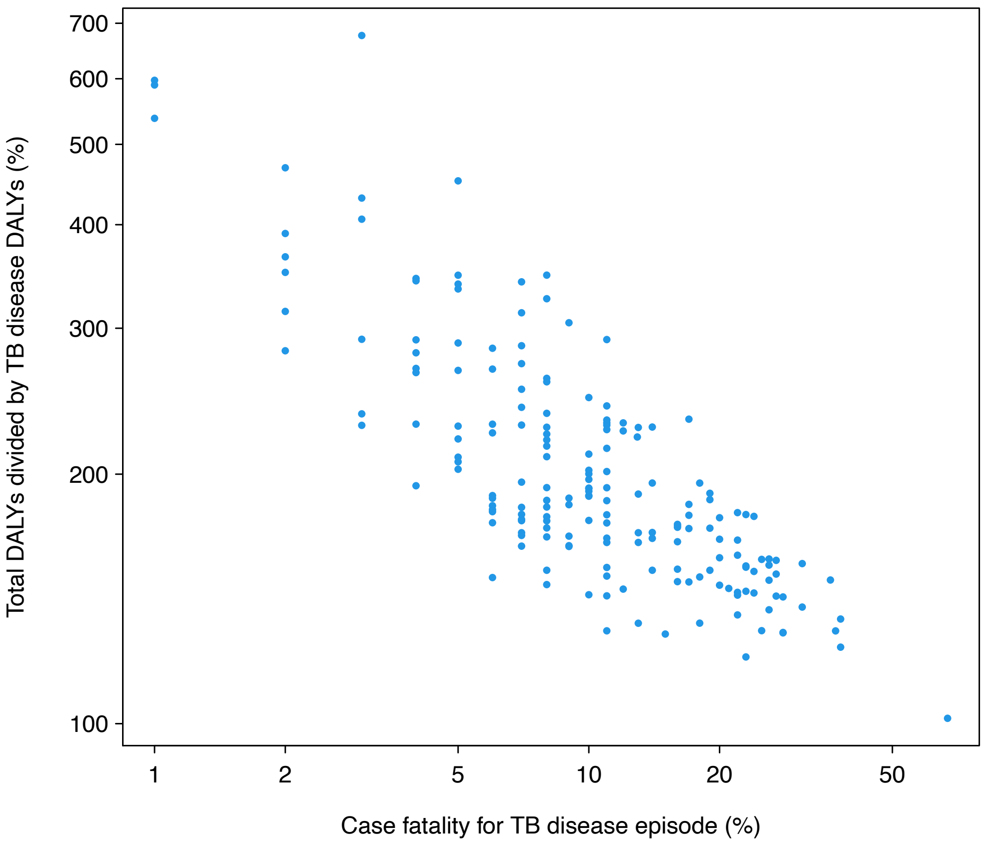

Figure S5: Country-level values for the case fatality of the TB disease episode plotted against the increase in total DALYs due to the inclusion of post-TB, log-log projection.

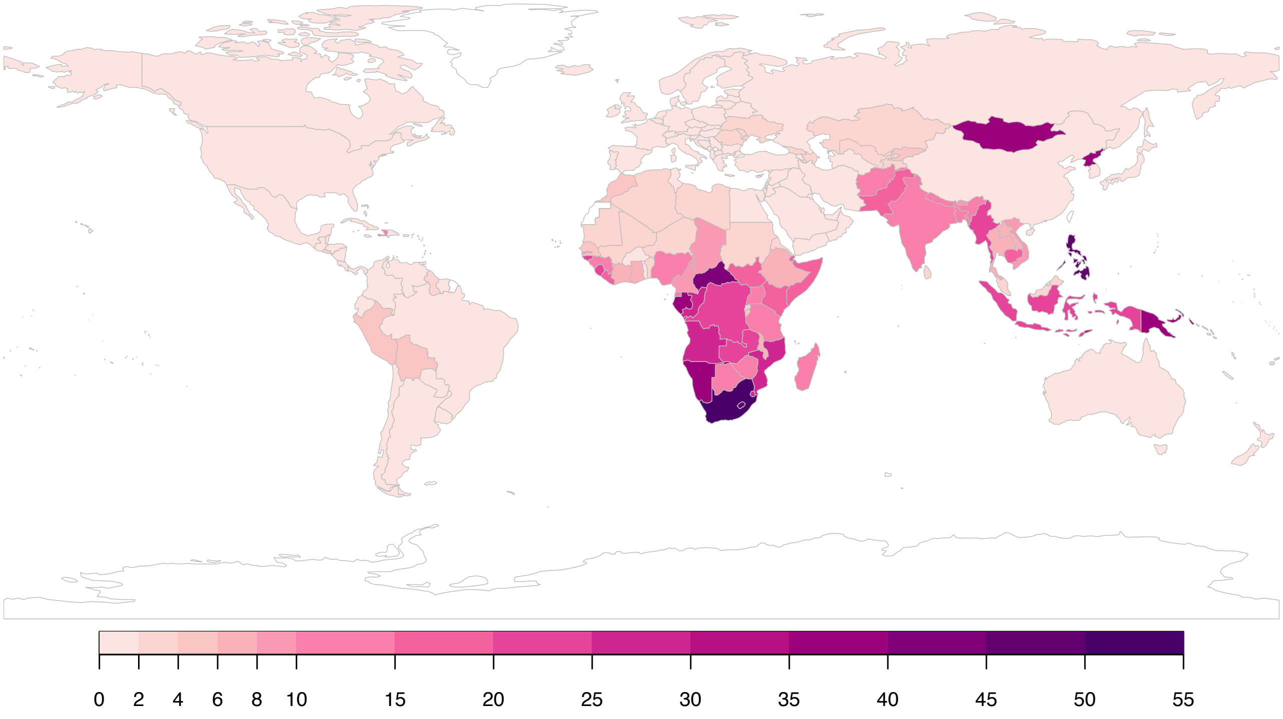

Figure S6: Global distribution of post-TB DALYs per 1000, attributable to incident TB disease in 2019.

Country	TB disease	Post-TB	Total	Percent increase with post-TB (%)
Angola	0.88 (0.75, 1.03)	0.83 (0.51, 1.24)	1.72 (1.36, 2.17)	95 (57, 146)
Bangladesh	1.48 (1.25, 1.74)	1.92 (1.27, 2.79)	3.40 (2.66, 4.35)	131 (82, 192)
Brazil	0.25 (0.20, 0.30)	0.34 (0.21, 0.51)	0.59 (0.44, 0.77)	141 (87, 219)
Cambodia	0.12 (0.10, 0.15)	0.29 (0.18, 0.43)	0.41 (0.30, 0.54)	241 (149, 365)
Central African Republic	0.36 (0.30, 0.42)	0.20 (0.11, 0.31)	0.56 (0.45, 0.68)	56 (31, 88)
China	1.18 (0.94, 1.51)	2.86 (1.87, 4.13)	4.05 (2.95, 5.42)	244 (152, 368)
Congo	0.23 (0.20, 0.27)	0.14 (0.08, 0.20)	0.37 (0.30, 0.45)	58 (35, 89)
Democratic People's Republic of Korea	0.78 (0.66, 0.91)	0.94 (0.53, 1.45)	1.72 (1.28, 2.24)	122 (68, 192)
Democratic Republic of the Congo	2.45 (2.08, 2.85)	1.88 (1.17, 2.79)	4.32 (3.49, 5.36)	77 (48, 117)
Ethiopia	1.18 (1.00, 1.37)	0.87 (0.60, 1.26)	2.05 (1.68, 2.47)	74 (49, 108)
India	20.65 (17.51, 24.03)	14.79 (9.86, 21.20)	35.44 (29.07, 43.29)	72 (46, 105)
Indonesia	3.98 (3.37, 4.68)	5.62 (3.44, 8.43)	9.60 (7.24, 12.57)	142 (87, 215)
Kenya	1.69 (1.44, 1.97)	0.88 (0.57, 1.29)	2.57 (2.14, 3.10)	53 (33, 78)
Lesotho	0.20 (0.17, 0.24)	0.10 (0.06, 0.15)	0.30 (0.25, 0.36)	49 (27, 79)
Liberia	0.17 (0.14, 0.20)	0.10 (0.06, 0.14)	0.26 (0.22, 0.32)	58 (35, 87)
Mozambique	0.46 (0.39, 0.53)	0.87 (0.53, 1.29)	1.32 (0.96, 1.75)	191 (115, 290)
Myanmar	0.96 (0.82, 1.13)	1.22 (0.75, 1.83)	2.19 (1.68, 2.82)	128 (77, 194)
Namibia	0.12 (0.10, 0.14)	0.09 (0.05, 0.14)	0.21 (0.17, 0.26)	79 (45, 124)
Nigeria	8.30 (6.97, 10.04)	2.42 (1.60, 3.52)	10.73 (9.04, 12.69)	29 (19, 44)
Pakistan	1.51 (1.23, 1.86)	3.71 (2.36, 5.44)	5.21 (3.81, 6.97)	248 (153, 375)
Papua New Guinea	0.26 (0.22, 0.30)	0.33 (0.19, 0.50)	0.59 (0.44, 0.76)	126 (72, 195)
Philippines	1.41 (1.15, 1.71)	4.91 (2.76, 7.53)	6.32 (4.13, 8.93)	352 (190, 557)
Russian Federation	0.39 (0.33, 0.46)	0.26 (0.17, 0.39)	0.66 (0.53, 0.80)	67 (44, 102)
Sierra Leone	0.14 (0.12, 0.16)	0.18 (0.11, 0.26)	0.32 (0.24, 0.40)	128 (80, 193)
South Africa	2.38 (2.01, 2.82)	3.15 (1.76, 4.85)	5.53 (4.07, 7.24)	133 (73, 213)
Thailand	0.35 (0.30, 0.41)	0.46 (0.31, 0.67)	0.81 (0.64, 1.03)	132 (87, 191)
United Republic of Tanzania	1.57 (1.34, 1.83)	0.76 (0.50, 1.10)	2.33 (1.95, 2.79)	49 (31, 73)
Viet Nam	0.40 (0.33, 0.49)	0.85 (0.57, 1.22)	1.25 (0.95, 1.64)	213 (135, 315)
Zambia	0.77 (0.65, 0.89)	0.39 (0.24, 0.58)	1.16 (0.97, 1.39)	52 (32, 79)
Zimbabwe	0.28 (0.24, 0.32)	0.18 (0.12, 0.26)	0.46 (0.38, 0.56)	67 (44, 97)

Table S1: Estimated DALYs for TB disease and post-TB (millions), for the thirty high-TB burden countries, 2019.

Country grouping	Reductions in life-years lived by disease stage (millions) TB	Post-TB	Total	Percent increase with post-TB (%)*
Eastern Mediterranean	1.16 (0.98, 1.38)	1.49 (0.96, 2.20)	2.65 (2.03, 3.46)	129 (86, 191)
Europe	0.42 (0.36, 0.49)	0.27 (0.17, 0.42)	0.69 (0.55, 0.85)	65 (42, 97)
Africa	10.81 (9.09, 12.91)	4.94 (3.07, 7.56)	15.75 (12.75, 19.67)	46 (29, 68)
Western Pacific	1.45 (1.23, 1.72)	3.21 (2.08, 4.76)	4.66 (3.45, 6.33)	222 (149, 328)
Americas	0.40 (0.34, 0.46)	0.39 (0.26, 0.58)	0.79 (0.62, 1.00)	99 (66, 147)
South-East Asia	14.81 (12.60, 17.53)	8.29 (5.31, 12.37)	23.10 (18.83, 28.84)	56 (37, 83)
WHO High-Burden	25.21 (21.35, 29.96)	16.62 (10.55, 24.88)	41.83 (33.62, 52.75)	66 (43, 98)
**Global**	29.04 (24.59, 34.51)	18.60 (11.99, 27.61)	47.63 (38.46, 59.72)	64 (42, 95)

Table S2: Total reductions in life-years lived as a result of incident TB in 2019, globally and by world region.

* Compares outcomes with vs. without the inclusion of post-TB.

Outcome	TB disease	Post-TB	Total	Percent increase with post-TB (%)
		Main analysis results		
YLLs	59.3 (50.6, 69.6)	42.1 (28.3, 59.8)	101.4 (83.6, 122.8)	71 (47, 104)
YLDs	4.5 (2.2, 8.0)	15.6 (8.6, 24.4)	20.2 (12.5, 29.5)	383 (154, 755)
DALYs	63.8 (53.9, 75.2)	57.8 (38.4, 83.0)	121.6 (98.3, 151.2)	91 (59, 133)
Reduction in life-years lived	29.0 (24.6, 34.5)	18.6 (12.0, 27.6)	47.6 (38.5, 59.7)	64 (42, 95)
		Alternative specification with higher mortality risk ratio for post-TB		
YLLs	59.3 (50.6, 69.6)	94.1 (78.7, 111.6)	153.3 (132.2, 177.1)	159 (130, 191)
YLDs	4.5 (2.2, 8.0)	15.6 (8.6, 24.4)	20.2 (12.5, 29.5)	383 (154, 755)
DALYs	63.8 (53.9, 75.2)	109.7 (92.5, 130.4)	173.5 (150.1, 200.0)	173 (142, 208)
Reduction in life-years lived	33.3 (28.2, 39.4)	49.6 (39.6, 60.8)	82.9 (69.4, 98.1)	149 (121, 180)

Table S3: Global burden of disease outcomes for TB disease and post-TB due to incident TB in 2019, comparing results from the main analysis an alternative specification with a higher mortality risk ratio for post-TB.

Parameter	Total Global DALYs by disease stage: TB disease	Post-TB	Total	Percent increase with post-TB (%)*
Odd ratio for respiratory disease with post-TB	-0.01 (-0.07, 0.05)	0.86 (0.82, 0.89)	0.83 (0.79, 0.86)	0.85 (0.82, 0.88)
Total global TB cases in 2019	0.89 (0.87, 0.92)	0.46 (0.41, 0.52)	0.70 (0.66, 0.75)	0.01 (-0.05, 0.07)
Average TB case fatality rate	0.89 (0.86, 0.92)	-0.09 (-0.15, -0.03)	0.38 (0.33, 0.44)	-0.49 (-0.55, -0.44)
Mortality rate ratio for FEV1% below 80%	0.01 (-0.06, 0.07)	0.28 (0.22, 0.34)	0.26 (0.20, 0.32)	0.27 (0.21, 0.33)
Disability weight for moderate COPD	0.06 (0.00, 0.12)	0.26 (0.20, 0.32)	0.23 (0.17, 0.29)	0.27 (0.21, 0.33)
Duration of treated TB	0.55 (0.50, 0.60)	0.03 (-0.03, 0.09)	0.18 (0.12, 0.25)	-0.14 (-0.20, -0.08)
Fraction of TB untreated in 2019	0.59 (0.54, 0.64)	-0.02 (-0.08, 0.04)	0.18 (0.12, 0.24)	-0.20 (-0.26, -0.14)
Duration of untreated TB	0.38 (0.32, 0.44)	-0.01 (-0.08, 0.05)	0.09 (0.03, 0.15)	-0.09 (-0.16, -0.03)
Disability weight for TB	0.39 (0.34, 0.45)	-0.07 (-0.13, 0.00)	0.05 (-0.01, 0.11)	-0.17 (-0.23, -0.11)
Mortality rate ratio for post-TB individuals	0.04 (-0.02, 0.10)	-0.04 (-0.10, 0.02)	-0.05 (-0.11, 0.02)	-0.03 (-0.10, 0.03)
Disability weight for mild COPD	-0.02 (-0.08, 0.05)	0.04 (-0.02, 0.10)	0.04 (-0.02, 0.10)	0.04 (-0.02, 0.11)
Disability weight for severe COPD	-0.03 (-0.09, 0.03)	0.06 (0.00, 0.12)	0.04 (-0.02, 0.10)	0.08 (0.02, 0.14)
Duration of treated TB, with HIV	-0.02 (-0.08, 0.04)	-0.04 (-0.10, 0.03)	-0.04 (-0.10, 0.02)	-0.04 (-0.10, 0.03)
Disability weight for TB and HIV	0.04 (-0.02, 0.10)	0.01 (-0.05, 0.07)	0.02 (-0.05, 0.08)	-0.02 (-0.08, 0.05)
Duration of untreated TB, with HIV	-0.01 (-0.07, 0.05)	0.01 (-0.05, 0.07)	0.01 (-0.05, 0.07)	0.02 (-0.04, 0.08)
Disability weight for symptomatic HIV, no ART	0.04 (-0.02, 0.10)	0.01 (-0.05, 0.07)	0.00 (-0.06, 0.06)	0.02 (-0.05, 0.08)
Disability weight for HIV on ART	0.02 (-0.05, 0.08)	0.00 (-0.06, 0.06)	0.00 (-0.06, 0.06)	-0.01 (-0.07, 0.06)

Table S4: Partial rank correlation coefficients (PRCCs) for four major outcomes (global DALYs for TB disease, post-TB, and both combined, and the percentage increase in total DALYs with the inclusion of post-TB)*.

* Inputs ordered by magnitude of PRCC for total DALYs (column 3).
